# Insights into Public Health Policy and Practice: The Role of Social Determinants in Mental Health and Resilience After Disasters

**DOI:** 10.1192/j.eurpsy.2023.2046

**Published:** 2023-07-19

**Authors:** W. Mao, V. Agyapong

**Affiliations:** 1 Psychiatry, University of Alberta, Edmonton; 2 Psychiatry, Dalhousie University, Halifax, Canada

## Abstract

**Introduction:**

Both natural disasters such as wildfires, earthquakes, tsunamis, and hurricanes, as well as man-made disasters such as civil wars, have been known to result in significant mental health effects on their victims.

**Objectives:**

The purpose of this general literature review is to analyze the impact and contribution of social determinants to mental health and resilience following natural and man-made disasters.

**Methods:**

In this paper, we specifically explore some of the most studied factors relating to vulnerability and protection, such as gender, age, ethnicity, social support, and socioeconomic status on mental health and resiliency in disaster survivors. In addition, several other possible factors were discussed, such as previous trauma, childhood abuse, family psychiatric history, and subsequent life stress.

**Results:**

Using key words such as mental health, social determinants, disasters, wildfires, earthquakes, terrorism attacks, and resilience, we conducted a literature search in major scientific databases

**Conclusions:**

A discussion of the implications for public health policy and practice is presented

**Disclosure of Interest:**

None Declared

